# Supplementary material for: Pestilent relationship between smoking and hypertension or pulse pressure among males over 15 years in India: NFHS-5 Survey
Source: PLoS One. 2024 May 3;19(5):e0294898. doi: 10.1371/journal.pone.0294898 (PMC11068163; doi:10.1371/journal.pone.0294898)
Supplement: Attachment — Submitted filename: Response to Reviewers.docx [file pone.0294898.s003.docx]

27^th^ July 2023

To

The Editor

Plos One Journal

Respected sir/madam

I am writing this letter to express my heartfelt gratitude for your invaluable efforts and constructive feedback on our manuscript titled, “**Pestilent relationship between smoking and hypertension or pulse pressure among males over 15 years in India: NFHS-5 Survey**" submitted to Plos One. Your expertise and dedication have played a pivotal role in enhancing the quality and rigor of our work, and we are genuinely grateful for your time and commitment to this process.

Your insightful comments and thoughtful suggestions have been immensely valuable in refining our study and addressing its limitations. Your keen attention to detail and perceptive observations have challenged us to critically analyze our research, leading to significant improvements in the manuscript.

We deeply appreciate the thoroughness of your evaluation and the time you have taken to carefully review our work. Your expert guidance has been instrumental in shaping the manuscript into a more robust and scholarly contribution to the field.

We have carefully considered each of the points raised in your evaluation and have addressed them comprehensively in the revised manuscript. Below, we present our responses to each of your comments:

| **S No** | **Comments** | **Responses** |
| --- | --- | --- |
| 1. | Please ensure that your manuscript meets PLOS ONE's style requirements, including those for file naming. | The manuscript has been revised as per the guidelines issued by the journal. |
| 2. | Please include a complete copy of PLOS’ questionnaire on inclusivity in global research in your revised manuscript. Our policy for research in this area aims to improve transparency in the reporting of research performed outside of researchers’ own country or community. | A complete copy of PLOS’ questionnaire on inclusivity in global research has been included |
| 3. | PLOS requires an ORCID iD for the corresponding author in Editorial Manager on papers submitted after December 6th, 2016. Please ensure that you have an ORCID iD and that it is validated in Editorial Manager. | Included |
| 4. | Your ethics statement should only appear in the Methods section of your manuscript. If your ethics statement is written in any section besides the Methods, please delete it from any other section. | The ethics statement has been removed from all other places except for the Methods section. |
| 5. | We note that Figure 1 in your submission contain [map/satellite] images which may be copyrighted. All PLOS content is published under the Creative Commons Attribution License (CC BY 4.0), which means that the manuscript, images, and Supporting Information files will be freely available online, and any third party is permitted to access, download, copy, distribute, and use these materials in any way, even commercially, with proper attribution. For these reasons, we cannot publish previously copyrighted maps or satellite images created using proprietary data, such as Google software (Google Maps, Street View, and Earth). | Fig 1 is a Bing Map under Microsoft which gives general rights for prints under **Section 3 (Use with a Bing Maps Agreement), Section 4 (Use with a Volume Licensing Agreement) and Section 12 (Bing Maps API for Enterprise offerings purchased through the Azure Marketplace) of the TOU.**  **The full print rights are available online at the following link:**  <https://www.microsoft.com/en-us/maps/product/print-rights> |
|  | Additional Editor’s comments |  |
| 1. | Please make the case stronger for why you are revisiting the association between smoking and blood pressure. The few studies cited 9,10,11 are not substantial - one was in a subset of post menopausal women, one is a 1986 paper...I would recommend instead to highlight the associations between smoker hypertensive and the social determinants variables that have been presented in the paper. This is much more useful to the current discourse and will be a contribution of this manuscript. | Essential revisions have been diligently implemented, and we have also updated a few citations to incorporate articles that offer further substantiation of the association between smoking and hypertension. |
| 2. | The discussion needs to include more on pulse pressure - what is the significance of pulse pressure, its biological plausibility etc. | The discussion section has undergone extensive and thorough revision, resulting in a comprehensive depiction and analysis of the pulse pressure findings and their implications. The revised manuscript now presents a detailed comparison of these findings with various studies available in the literature. |
| 3. | State wise variation is mentioned- UP having highest prevalence of the smoker hypertensive, can be discussed | The prevalence of smoking and hypertension in Uttar Pradesh (UP) has been thoroughly examined and elaborated in the revised manuscript. Additionally, a comprehensive comparison has been drawn between the current findings and the data from NFHS-4 to provide valuable insights into the trends and changes in smoking and hypertension prevalence over time in this region. |
| 4. | Methodology needs a section on analysis | Methodology section has been fully revised and analysis section has now been included. |
| 5. | Please be clear in the paper- you can only comment on associations not causality, so its not the effect of smoking on hypertension (discussion line 1) but only an association that is clear- the pathways are complex and we know they are not single variable dependant. | The same has been mentioned in the limitation part of the manuscript and first line of discussion portion has been edited accordingly. |
|  | Reviewer #1 Comments |  |
| 1. | Introduction: we need to make strong base why only males have been included in the present study, when we know smoking has more deleterious effects on females. | It has been discussed and included in the study participants section of Methods. |
| 2. | similarly why we have only considered smoking tobacco should be justified. | The idea of including tobacco smoking for the present study has been described and included in the Introduction part of the revised manuscript. |
| 3. | methods: methods are vaguely written. we can consider using the format used by the corresponding author in the previous studies | The Methods section of the manuscript has been meticulously revised, and subheadings have been thoughtfully incorporated to enhance clarity and organization. This thoughtful approach ensures that readers can easily navigate and comprehend the methodology employed in our study. |
| 4. | We need to reconsider as people taking medication for htn are not included in the sample selection process as per written methodology. | The questionnaire is a part of NFHS-5 which includes 3 subsets. |
| 5. | table 2 is very complex. we can have one top row to depict total counts. then, subsequently we can only depict weighted% to depict distribution of smokers as per HTN. Also, we can have separate tables for HTN, and pulse pressure to give equal weightage to the two | Table 2 has been thoughtfully redesigned to improve its comprehensibility and comparability. To reduce the number of tables and enhance the ease of comparison, the two subsets (hypertension and pulse pressure) have been amalgamated into a single table. This strategic consolidation allows for a more concise presentation of the data while ensuring that readers can readily assess and compare the various stages of hypertension and pulse pressure categories (Normal, Narrow, and Wide). |
| 6. | table 3: why separate odds have been calculated for narrow and wide PP, and if there is a clinical theory to support then it should be highlighted somewhere in early parts of the manuscript. | The revised manuscript now comprehensively expounds on the distinct clinical implications of Narrow Pulse Pressure (PP) and Wide Pulse Pressure, recognizing their disparate significance in cardiovascular health. Owing to these divergent implications, separate odds ratios (OR) have been meticulously calculated for each category. This approach not only elucidates the unique associations of Narrow PP and Wide PP with various factors but also facilitates a more nuanced understanding of their respective impacts on cardiovascular outcomes. |
| 7. | discussion: last line of 2nd para is very vague. it should be duly cited, or else it looks like a vague assumption. not much has been mentioned about the independent variables chosen in the study in the methods section. | Discussion portion has been meticulously revised. |
| 8. | association and biological plausibility to depict association between pulse pressure and smoking has not been highlighted much in the discussion. like why the reader should be interested in pulse pressure when studying smoking. what are its consequence, how it is different from hypertension scientifically. | The difference and health implications of Pulse pressure has been described in the manuscript. |
| 9. | you can consider rewriting your discussion | Discussion part has been fully revised |
| 10 | While revising your submission, please upload your figure files to the Preflight Analysis and Conversion Engine (PACE) digital diagnostic tool, https://pacev2.apexcovantage.com/. | Editing using PACE has been done. |

In conclusion, we are grateful for the opportunity to address the issues raised by the academic editor and reviewer. We are confident that the revisions made in response to your invaluable feedback have significantly improved the manuscript's quality and scientific rigor.

Once again, we extend our sincere gratitude for your meticulous evaluation, which has undoubtedly strengthened the scholarly value of our work.

Thanking you

Yours sincerely

Authors
